# Ticks’ tricks: immunomodulatory effects of ixodid tick saliva at the cutaneous tick-host interface

**DOI:** 10.3389/fimmu.2025.1520665

**Published:** 2025-03-27

**Authors:** Lisa Kleissl, Sophie Weninger, Florian Winkler, Margarida Ruivo, Michiel Wijnveld, Johanna Strobl

**Affiliations:** ^1^ CeMM Research Center for Molecular Medicine of the Austrian Academy of Sciences, Vienna, Austria; ^2^ Department of Dermatology, Medical University of Vienna, Vienna, Austria; ^3^ Institute for Hygiene and Applied Immunology, Center for Pathophysiology, Infectiology and Immunology, Medical University of Vienna, Vienna, Austria

**Keywords:** tick bite, pathogen transmission, tick-borne diseases, Lyme borreliosis, tick saliva, parasite-host interface, immunomodulation, immune evasion

## Abstract

Due to changes in global climate, the geographic distribution of ticks and tick-borne infections is increasing and represents a growing global health concern for humans. Ticks of the genus Ixodidae are globally abundant and transmit a wide variety of pathogens that cause human infections, including tick-borne encephalitis and Lyme borreliosis. The transmission of pathogens into human skin while blood feeding causes changes in the local immune cell network and has various effects on structural skin cells, including sensory neurons. Recent studies have focused on the effect of tick saliva on cells at the cutaneous tick-host interface and have suggested a strong immunomodulatory function. Within seconds after a tick bite, saliva containing various bioactive molecules is secreted into the host’s skin, leading to vasodilation, inhibition of coagulation and anti-inflammatory actions. Inhibition of immune cell recruitment and cytokine secretion, facilitate prolonged tick attachment and blood feeding as well as pathogen transmission. Therefore, in recent years, efforts have intensified to identify tick salivary compounds by multi-omics approaches and investigate their individual effects on innate and adaptive immunological mechanisms. In this review, we summarize important features of tick saliva molecules and how they influence and modulate skin cell behavior on the tick-host interface to facilitate tick attachment and pathogen transmission. Further, we highlight immunomodulatory mechanisms of salivary compounds and their potential role as novel treatment agents for inflammatory skin diseases and in tick vaccine development.

## 1 Introduction

### 1.1 Basics of ticks as ectoparasites and disease vectors

Ticks are globally distributed parasitic arthropods, which are important vectors to multiple pathogens. Due to climate change, the abundance of ticks constantly increases worldwide (1). Ticks comprise about 900 different species, of which roughly 700 belong to the family of Ixodidae (hard ticks) (2–4). Ticks of the Ixodidae family are globally abundant arthropods relevant in transmitting pathogens, which are dangerous to humans and are, therefore, the focus of this review.

The Ixodidae family comprises different tick genera with more than 700 species, including genera *Amblyomma*, *Boophilus*, *Dermacenor*, *Haemaphysalis*, *Hyalomma*, *Rhipicephalus, and Ixodes* (2). The genus *Ixodes* consists of a complex of closely related tick species most relevant in transmitting pathogens to humans in Europe (3) causing a wide array of human diseases, including tick-borne encephalitis (4, 5), Lyme borreliosis (6), rickettsiosis (7), human granulocytic anaplasmosis (8), and babesiosis (4, 9). Members of the *Ixodes*complex are prevalent throughout the northern hemisphere (6, 10, 11).In Europe, *I. ricinus* functions as the most prominent vector (12), followed by *I. persulcatus* and *I. inopinatus*. The latter tick species has recently been described (13) and is widely distributed in Europe. Both *I. ricinus* and *I. inopinatus* share many morphological features and might have been misidentified in the past, especially as they can inhabit the same geographical areas (14, 15). Potential misidentification must be considered when reviewing data on tick-borne pathogen prevalence within *Ixodes* populations in Europe.

Humans are at the greatest risk of acquiring a tick-borne illness when they spend time in nature, unintentionally becoming hosts for ticks while engaging in recreational activities that overlap with tick habitats (6, 9, 10). While the original habitat of *Ixodes* species is wooded areas, it expanded to urban spaces due to the urbanization of forests and the focus on introducing green zones, increasing the risk of ticks and tick-borne pathogens in urban recreational areas (16–18). Contrary to popular belief, *Ixodes* ticks do not fall from trees but await passing hosts on low vegetation, including shrubs and grasses (19).

The life cycle of *Ixodes* includes four distinct stages: After the egg stage, a tick cycles through the developmental stages of larva, nymph, and finally reaches the adult stage, a process which typically takes 4-6 years and in warmer regions 2-3 years (20). As ticks are hematophagous, their development depends on blood meals from a host. However, the feeding period only lasts a few days during each developmental stage, and ticks live mostly non-parasitic (i.e., without being attached to a host) (20, 21). In the adult stage, a hard shield covers the entire dorsum of male and most of the dorsum of female Ixodidae. Four leg pairs arise from the tick's body in nymph and adult stages, while larvae only possess three leg pairs. The mouthparts of a tick, known as the capitulum, include a pair of chelicerae that can cut the skin, a hypostome for anchoring itself in the skin to feed on blood, and a pair of palps. *Brevirostrata* ticks including *Rhipicepahalus microplus* and *R. sanguineus* possess short mouthparts that only reach superficial layers of the host’s skin. In contrast, *Longirostrata* ticks such as the *Amblyomma cajennense* have a long mouthpart (22). To further increased stability for the feeding apparatus hard ticks secrete proteins and collagen, forming a cement cone (23) around the attachment site (20). The cone can vary in size and composition, depending on the anatomy of the mouthparts of different tick species and the number of hosts they feed on (22). Ingested blood reaches the tick’s midgut through the esophagus where it is stored in a pouch-shaped diverticula (24). Larvae and nymphs of *Ixodes* feed primarily on small mammals and may acquire pathogens, including *B. burgdorferi*, during these blood meals. Female ticks require more voluminous blood meals for reproduction and mainly feed on larger animals (11). Between bloodmeals, ticks carry pathogens in specific tissues of their body: The midgut, for instance, is a typical niche for *B. burgdorferi* to reside until further replication and translocation to the hemolymph and salivary glands during the subsequent bloodmeal (25). In addition, the tick’s gut microbiome influences the mucus-like surface of the midgut and hence contributes to the colonization of ticks by pathogens (3, 26). In addition to this classical model of tick colonization and pathogen transmission, recent research has suggested the regurgitation of microbes directly from the midgut into the host without the need for tick saliva (27). Further studies are required to confirm the role of this regurgitation model in the transmission dynamics of tick-borne pathogens.

As the main vectorial fluid during tick-borne pathogen transmission in the classical transmission model, tick saliva has been a focus of an abundance of studies. Within seconds of attaching to its host, a tick secretes saliva into the human skin. This process persists throughout the blood meal and lasts up to two weeks, depending on the life stage and tick species. During this time, ticks must bypass or suppress the host’s immune mechanisms to enhance feeding efficiency and remain undetected. This is accomplished by a vast number of bioactive components present in tick saliva, which allow ticks to modulate the hosts’ immune system and achieve vasodilation, anticoagulation, as well as inhibition of complement activation and platelet aggregation (28–31). Besides the above-described functions, certain saliva molecules, including lipocalins, can counteract sensations of pain and itch. Therefore, tick bites stay mostly unrecognized, and ticks can remain firmly attached to the skin undisturbed (32–34). Interestingly, tick saliva was shown to be modified in its composition by pathogens to enhance their survival and transmission rates (35). The subsequent sections focus on production, contents, and functions of tick saliva in more detail.

### 1.2 Tick saliva and salivary glands

Tick saliva, produced in the salivary gland acini, is one of the most complex of all animals (31). Salivary glands are crucial organs for tick survival, host interaction, and pathogen transmission. Salivary secretion begins in tick salivary glands via a multitude of molecular signaling pathways (36–39). The neurotransmitter dopamine has been identified as a key player in this process, activating receptors in glands to initiate cAMP- and calcium-dependent pathways (40, 41). There are three different types of acini in female and five different in male ixodid ticks, that contribute to *de novo* saliva production as well as storage of saliva compounds in granules (31, 42, 43). Type I acini are crucial for tick survival in off-host periods, harboring components of the water vapor uptake preventing dehydration (42, 44). Salivation is an important mechanism for maintaining tick homeostasis and body size. It allows to correct fluid imbalance and hydrostatic pressure within the tick, alternating between uptake and secreting every 5-30 seconds (42, 45). During feeding, type II and III acini of ixodid females expand in mass and size and regulate uptake of water and electrolytes from the surrounding hemolymph via their epithelium and secret excess fluid back into the host (44, 46–48). In addition, type II acini of ixodid species harbor cement cells facilitating cement secretion. Tick cement is a matrix consisting of fibrils and sheets with a high content of tick- and host-derived proteins including glycine rich proteins, metalloproteases, and protease inhibitors. Cement cones, harboring various bioactive molecules, are important for the proper fixation of the mouthparts inside the host’s skin and at the same time provide protection from the host’s immune system (42, 43, 46, 49).

**Figure 1:**
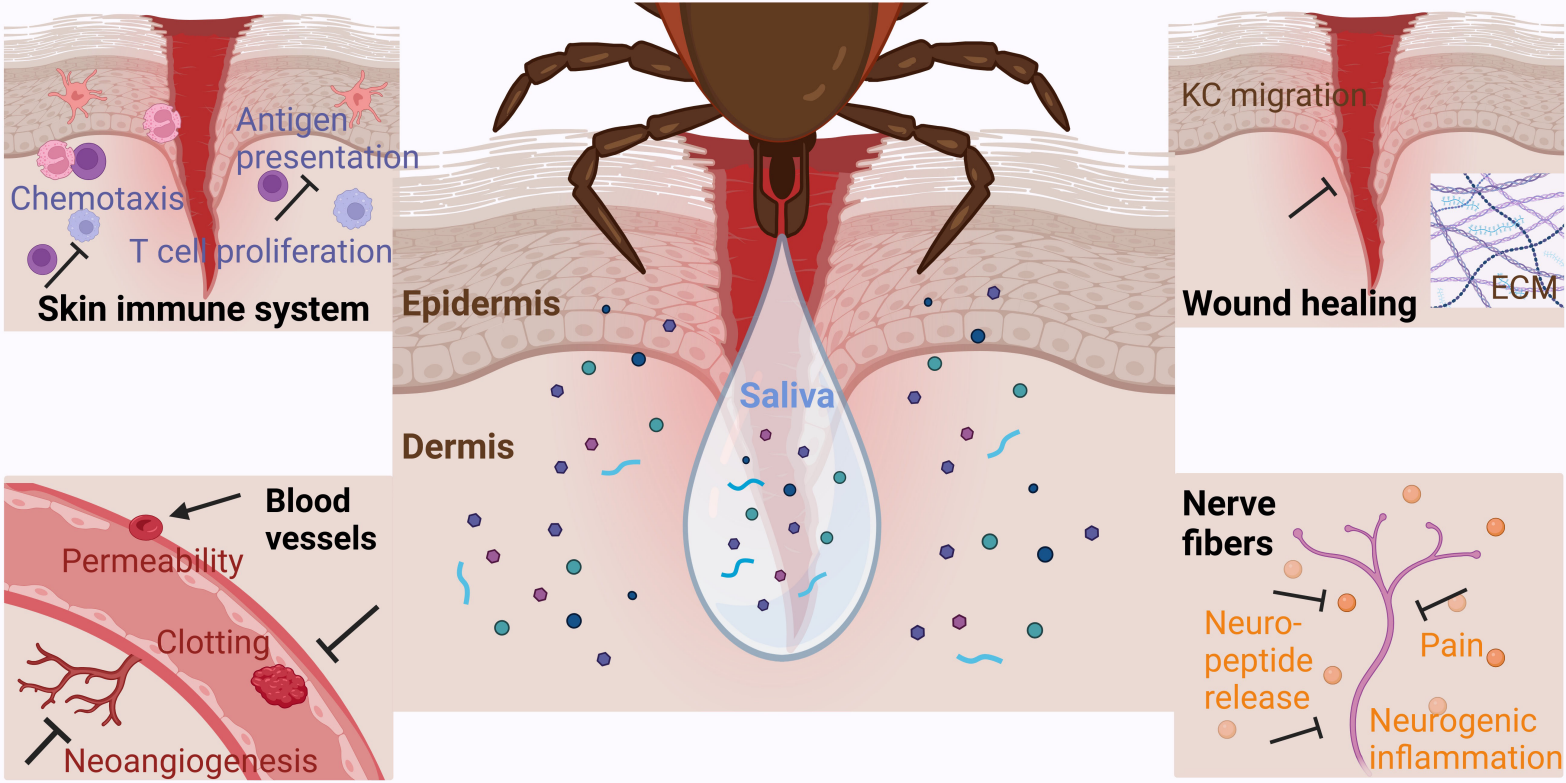
Classes of tick saliva components in ixodid species.

Tick saliva mostly consists of water and ions derived from the bloodmeal. Its bioactivity is generated by a mixture of proteins, extracellular vesicles, peptides as well as non-peptide molecules (**Figure 1**) (31). Studies identified a multitude of saliva constituents from various tick species and during different feeding stages using sequencing and proteomic approaches (50–55). Various tick saliva proteins exhibit immunomodulatory functions (**Table 1**). This includes several protein families: serpins, a large family of serine protease inhibitors; cystatins, reversible inhibitors of cysteine proteases; evasins, glycoproteins inhibiting mammalian chemokines; *I. scapularis* salivary proteins (Salp proteins); lipocalins, a protein family with a characteristic structure including a binding pocket for hydrophobic substances; metalloproteases, and Kunitz domain-containing proteins, also acting as serine protease inhibitors (56–60). Many of the protein families are shared between different tick species, but the exact sialome composition varies according to tick family, feeding time point, and is depending on the host. In addition, host proteins are filtered from the blood meal, pass the midgut, hemocoel and salivary glands before they are secreted within the saliva (61, 62). However, not all host proteins are recycled into the saliva; instead, it is thought that there are selective mechanisms that differentially return or concentrate proteins within the saliva. For example, tick proteins Salp15 (63) and tick salivary lectin pathway inhibitor (TSLP) (64) were identified to interact with host proteins to influence tick and larvae infestation as well as pathogen survival within the tick, causing higher transmission rates to new hosts (65). However, selective recycling of host proteins into tick saliva is incompletely understood and remains an area for further research.

**Table 1:** Protein families with known biological functions in tick saliva.

Biological function	Protein family	Examples
Anticoagulants	Enzymes	Apyrase, 5’-Nucleotidase
	Kunitz-domain containing proteins	Ixolaris, Tissue factor pathway inhibitor (TFPI)-like proteins
	Lipocalins	Histamine and leukotriene-binding proteins
Chemokine and Cytokine modulators	Evasins	Evasin 1, 3, 4
	Serpins	Iris, IRS-2, Iripin-1
	Cystatins	Sialostatin L, Sialostatin L2
	Lipocalins	Histamine and leukotriene-binding proteins
	Enzymes	DNases/RNases, Prostaglandin E2 synthase, Adenosine deaminase-like proteins
Complement Inhibitors	I. scapularis salivary proteins	ISAC, Salp-20
	I. ricinus salivary proteins	IRAC I and II
	Serpins	Iripin-5 and 8
Structural proteins	Cement proteins	Glycine- and proline-rich proteins
	Mucin-like proteins	
Host interaction proteins	Tick receptor	Tick receptor for OspA (TROSPA)
	I. scapularis salivary proteins	Salp15
Miscellaneous Families	Antimicrobial peptides	
	Heat shock proteins	

Both host- and tick-derived proteins are comprised in the TickSialoFam database (66). It categorizes numerous putative secreted proteins based on Position-Specific Iterative Basic Local Alignment Search Tool (PSI-BLAST, https://blast.ncbi.nlm.nih.gov), a scoring matrices-based method for detection of relationships between protein sequences. The authors generated seven functional groups, including enzymes, protease inhibitors, tick-specific anti-coagulative peptides, immune-related proteins and antimicrobials. Overall, approximately 300,000 distinct protein sequences found in tick saliva are listed in the TickSialoFam database. While the list of tick salivary compounds and their annotated molecular and putative function is still expanding, many of them have already been comprehensively reviewed (28, 67–70). Here, we will focus on the compounds that influence successful tick feeding, the immune composition and hemostatic defense at the cutaneous tick-host interface.

## 2 Changes in the cutaneous immune network in response to tick saliva

As an effective blood meal on a host is achieved when the tick remains unnoticed for several days, it is plausible that ticks employ evolutionary acquired mechanisms to influence cutaneous immune cell composition to counteract the host’s immune response, hemostasis, and wound healing. To this end, ticks employ various strategies in which bioactive components of their saliva play a critical role in circumventing recognition by the host (71).

The early cellular immune response in the human skin due to an *I. ricinus* tick bite is characterized by the predominance of neutrophils, macrophages and dendritic cells, which migrate to the skin in response to increased levels of chemoattractants, including chemokine ligand (CCL)-2, CCL3, CCL4 and C-X-C motif chemokine ligand (CXCL)-1. Conversely, lymphocyte number and local levels in lymphocyte chemoattractants remain unchanged and are comparable to healthy skin (72). Several hours after exposure to tick saliva, a decrease in antigen-presenting cells, especially dermal dendritic cells and Langerhans cells, and an increase in tissue-resident memory T cells and B cells can be observed in skin adjacent to the previous bite site. A tick bite can even induce detectable alterations in immune cell composition within the circulation, characterized by a slim decrease in T cell and natural killer cell numbers in the peripheral blood (73).

The subsequent sections summarize how the multitude of tick saliva proteins modulate specific mechanisms of the host’s immune system at the cutaneous interface, facilitating and enhancing ectoparasite survival.

### 2.1 Modulation of the innate immune system and inhibition of chemoattraction by tick feeding

The innate immune system provides a rapid first line of defense to host infection as it is not dependent on prior antigen exposure. As the first minutes after a tick bite are key for successful tick attachment and blood feeding, it is essential for tick survival to suppress its host innate immune system within minutes and therefore evade tick rejection (74). Therefore, following a tick bite and contact with tick saliva, the cutaneous and circulatory immune networks undergo dramatic, rapidly occurring changes (**Table 2**) (73, 75).

**Table 2:** Examples of tick saliva molecules with immunomodulatory functions.

Category	Molecule	Function
Inhibition of T-cell activity	Salp15	Interferes with T cell receptor (TCR) signaling, IL-2 production, activation of CD4+ T cells by dendritic cells (117, 128).
	Sialostatin L	Inhibits cathepsin L, reducing antigen presentation and T cell activation (143). Inhibits production of IL-9 in mast cells and maturation of dendritic cells
Complement Inhibition	Salp20	Inhibits the alternative complement pathway, protecting pathogens from complement attack (58).
	ISAC	Negative regulator of alternative complement activation (104).
	IRAC I and II	Negative regulator of alternative complement activation (105).
Anti-inflammatory function	Histamine-binding proteins (HBP)	Neutralize histamine to suppress local inflammation (144).
	Prostaglandin E2	Modulates inflammatory responses and promotes vasodilaton (92). Inhibits T cell activation.
	Sialostatin L2	Reduces pro-inflammatory cytokine production (e.g., TNF-α, IL-12) by targeting cathepsins (143). Inhibits NLRC4-mediated inflammasome activation.
Anti-coagulant	Ixolaris	Inhibits thrombin generation and blood coagulation, supporting efficient blood-feeding (145).
	Serpins	Serin protease inhibitors regulating angiogenesis, inflammation, and tissue remodeling (146).
Immune cell recruitment	Tick salivary lectin pathway inhibitor (TSLP)	Impairs neutrophil phagocytosis and chemotaxis (64).
	Apyrase	Hydrolyzes ATP and ADP, inhibiting neutrophil activation and platelet aggregation (147).
	Evasins	Bind and neutralize host chemokines, effectively suppressing immune cell recruitment (98).

Neutrophils are recruited immediately to the skin in response to injury and infection and cause an early inflammatory response (76). Once the tick mouthparts penetrate the host skin and trigger a cellular response, neutrophils and other innate immune cells are frequent targets of immunosuppressive molecules in tick saliva, mainly cystatins and serpins. By targeting cathepsin L and C, which are considered key proteases involved in host immune responses, cystatins modulate inflammatory cell migration. By using a carrageenan-induced paw edema model, Kotsyfakis et al. could demonstrate that sialostatin L, derived from *I. scapularis*, inhibits neutrophil infiltration on the basis of the above mentioned mechanism of action (77). In a mannan-induced psoriasis model, Wu et al. were able to confirm the inhibitory effect of sialostatin L on neutrophil infiltration. However, this effect was not limited to sialostatin L. Other cystatins, including mialostatin, sialostatin L2 and iristatin also led to an inhibition of neutrophil infiltration (78). The detailed function of the latter is described below. Serpins are a group of serine protease inhibitors with a molecular weight of 40 to 60 kDa, harboring a serpin reactive center loop to interact with the targeted protease, and are generally involved in the regulation of angiogenesis, inflammation, and tissue remodeling (79). They are secreted with tick saliva to block the host’s cutaneous innate immune cell infiltration and initial inflammatory response (79, 80). Various serpins have been identified in tick saliva of different species, and some were identified as strong immunomodulates, including *I. ricinus* immunosuppressor (Iris), *I. ricinus* serpin-2 (IRS-2) (81) and *Haemaphysalis longicornis* (Hl) Serpin-a and HlSerpin-b. *I. ricinus* serpin (Iripin)-1 is upregulated in the salivary glands of feeding ticks and was shown to attenuate neutrophil and monocyte recruitment to the tick attachment site by inhibiting the enzymatic activity of serin proteases involved in inflammation (trypsin, neutrophil elastase and kallikrein). Additionally, Iripin-1 reduced the expression of chemokine receptors on antigen-presenting cells and adhesion molecules on endothelial cells necessary for leucocyte transendothelial trafficking. At the same time, it increased the production of the anti-inflammatory cytokine IL-10, further promoting immunosuppression at the bite site (82). A molecule of the same family, Iripin-3, was shown to reduce the production of pro-inflammatory IL-6 by lipopolysaccharide (LPS)-stimulated bone marrow-derived macrophages (83), and mediated strong effects on adaptive immune cells as described below. In addition to cystatins and serpins, the chemokine-binding proteins evasins and presumably also complement inhibitory molecules derived from ticks inhibit the infiltration of neutrophils and other immune cells by blocking chemotaxis. The detailed effects of evasins and complement inhibitory molecules are described below.

Similarly, the pharmacoactive family of cystatins do not only modulate innate, but also adaptive immunity. In mice, Iristatin inhibits the proteolytic activity of cathepsin L and C and thereby blocks ROS production by macrophages, inflammatory cytokine secretion of mast cells and reduces IL-2, IL-4, IL-9 and IFN-γ production in T cells, which are essential for pathogen elimination and tick immunity (84). In a thioglycollate-induced peritonitis mouse model Iristatin treatment impaired overall immune cell recruitment to the peritoneum with the greatest impact on neutrophils and myeloid cells (84). Similarly, the newly identified secreted cystatin, Ricistatin of *I. ricinus* ticks exerts its immunomodulatory functions by a specific reactive center against cysteine cathepsins (85). The above mentioned sialostatin L, derived from *I. scapularis*, not only inhibits neutrophil infiltration at inflammatory sites, but also suppresses the maturation of dendritic cells (86). In addition, sialostatin L has been shown to inhibit the production of IL-9 in mast cells by interfering with IRF4 and IL-1β expression (87). Also *I. persulcatus*-derived sialostatin L1 and L2 were shown to inhibit bone marrow-derived dendritic cell maturation and production of IFN-γ–induced protein-10/CXCL10, TNFα, and IL-6 after LPS stimulation (88). During infection with pathogens like *Borrelia miyamotoi* and *Anaplasma phagocytophilum*, sialostatin L2 inhibits immune cell activation and cytokine production by splenic cells (89), as well as NLRC4 inflammasome activation in mouse bone marrow-derived macrophages by binding Annexin A (90). Ricistatin, a secreted type 2 cystatin identified in *I. ricinus*, significantly reduces the production of pro-inflammatory cytokines (TNF-α, IL-1β, IL-6) and reactive oxygen species in murine macrophages, suggesting suppression of proper myeloid function (85). Saliva compounds of *Amblyomma sculptum*, an important vector for *Rickettsia rickettsii*, the causative agent of Rocky Mountain spotted fever, were shown to interact with skin resident dendritic cells and impair their function (91, 92). Prostaglandin E2 (PGE2) contained in tick saliva was identified to inhibit secretion of pro-inflammatory TNF-α, IL-6, IL-1β and IL-12 after stimulation of murine and human dendritic cells and dampened humoral immunity to *Rickettsia* in mice (92). PGE2 derived from *I. dammini* has been shown to inhibit interleukin 2 production and T cell activation (87).

Among other ectoparasites, ticks can secrete structurally unrelated chemokine binding proteins, termed evasins. Two different classes of evasins can be distinguished according to their selectivity for CC or CXC chemokines, as well as a third group of evasin-like proteins (93). Evasin 1 and 4 solely bind to CC chemokines, whereas Evasin 3 binds to CXC chemokines, both harboring the potential to disrupt chemotaxis of activated immune cells (94, 95). Promptly after an ixodid tick bite, increased levels of chemotactic molecules and accumulation of antigen-presenting cells can be observed in human and murine tissue adjacent to, but not directly at, the bite site. Twenty-four hours after tick attachment the inflammatory response decreases (75, 96). This phenomenon might be partially explained by the secretion of evasins contained in the saliva and their attributed anti-chemotactic and anti-inflammatory properties in other, non-tick-transmitted pathologies (95, 97, 98). Evasin 1 and 3, derived from *Rhipicephalus sanguineus*, are both effective in blocking CCL3-dependent recruitment of dendritic cells after infection with *Leishmania major*, a pathogen transmitted by sandflies (*Phlebotomus* spp.), in otherwise resistant mice (99). Evasin 1 has the potential to reduce CCL3-dependent neutrophil recruitment in mouse models of lung fibrosis (100) and reversed skin inflammation in a psoriasis-like mouse model (60, 98). Evasin 3 possesses similar properties and may inhibit neutrophil chemotaxis by blocking chemokine ligand binding to CXCR2. Additionally, evasin 3 negatively influences lymphocyte recruitment to inflammatory tissues in murine models (60, 98). Evasin 4 was shown to reduce infarct size and leukocyte infiltration as well as circulatory levels of CCL2 in a post-myocardial infarction injury mouse model (60). These results highlight the suggested immunomodulatory effects of tick evasins during tick bites and their potential use to treat inflammatory diseases.

Complement activation is another key component of the early immune response against pathogens and serves as a coordinative link between serum and cellular response mechanisms by regulating proteolytic cascades via the classical, lectin and alternative pathway (101). Various host proteins are involved in regulating this process by either inhibiting or promoting complement activation at different stages. These tightly regulated processes of complement activation might be impaired by the mode of action of certain tick saliva molecules. Properdin, a protein activating the alternative complement pathway, was shown to be inhibited by a protein later termed complement inhibitor from hard tick *Rhipicephalus pulchellus* of the alternative pathway (CripA), thereby leading to decreased complement activation (102). OmCI, a broad-acting C inhibitory protein isolated from the soft tick *Ornithodoros moubata*, inhibits the alternative pathway of the human complement cascade by targeting C5 activation (103). Daix et al. characterized the *I. ricinus* anti-complement (IRAC) proteins I and II, which gene expression levels are upregulated in *I. ricinus* salivary glands during blood feeding and inhibit the complement system (104). IRAC I and II inhibit the alternative pathway of the human complement, similar to the *I. scapularis* salivary anticomplement (ISAC) protein, which functions as a regulator of complement activation like decay accelerating factor and factor H (104, 105). Hourcade et al. described the inhibitory effect of the glycoprotein Salp20 on the complement cascade in a mouse model: By synergism with the plasma protein factor H, Sapl20 counteracts the alternative activation of the complement system (58). Additionally, the salivary proteins Iripin-5 and Iripin-8 which belong to the family of serpins and are found in *I. ricinus* ticks, act as complement inhibitors (106, 107).

In addition to classical concepts of innate immune responses, the field of structural immunity gained a lot of attention in the last few years, highlighting the importance of structural cells, including keratinocytes and fibroblasts, in the initiation, guidance and modulation of immune responses (108, 109). There is recent evidence that keratinocytes might serve as targets for the immunosuppressive function of molecules in tick saliva. During incubation with *B. burgdorferi* s.l. bacteria or their outer surface protein C (OspC), the tick saliva protein Salp15 inhibited the secretion of pro-inflammatory agents like IL-8, MCP-1 and several antimicrobial peptides (defensins, cathelicidin, psoriasin) from keratinocytes (110) and provides protection against the complement-mediated killing of these bacteria (111). Furthermore, tick saliva-derived exosomes were shown to inhibit re-epithelialization and modulate gene expression profiles of keratinocytes in a wound healing model, suggesting a potential mechanism to prolong the blood feeding process. Furthermore, *Amblyomma maculatum* and *I. scapularis* saliva-derived exosomes induced the expression of inflammatory CXCL8 and TNF-α but decreased CXCL12, fibroblast growth factor (FGF) 7, and vascular endothelial growth factor A (VEGFA), known to promote wound healing, on human keratinocytes (112). The potential influence of tick saliva on other structural skin cells remains an open area of research.

### 2.2 Adaptive immune system modulation by tick saliva compounds and influence of tick feeding on dendritic cell - T cell crosstalk

After the initial response phase, ectoparasitic feeding should activate the adaptive immune system to facilitate proper pathogen clearance and memory formation. However, ticks display several mechanisms to circumvent their host’s adaptive response, prolong feeding time, and, in consequence, promote pathogen transmission. Only recently, we could show that skin biopsies of human tick feeding sites are dramatically altered not only regarding the innate but also the adaptive immune network. Increased numbers of B and T cells with a decreased CD4+/CD8+ T cell ratio and impaired T cell responses and cytokine secretion were detected at the site of tick bites compared to healthy skin (73). Both innate lymphoid cells and T cells showed increased expression of cytokines associated with type-2 immunity. Tick-induced skewing towards a T helper (Th)2 response is a commonly observed mechanism after tick feeding and during tick-borne pathogen transmission to mammal skin. Skewed Th2 polarization might represent a favorable condition for ticks to avoid rejection by the host and in addition facilitate enhanced pathogen transmission. It was shown that the suppression of Th2 cytokines reduces tick-transmitted *B. burgdorferi* load in mice and that Th1 polarization and concomitant IFN-γ production is essential for the acquisition of tick immunity by resistant hosts (113).

Several studies aimed at identifying the tick-derived agents (114–116) responsible for the numerous immunomodulatory effects on T cells. These include impaired T cell priming by antigen-presenting cells as well as direct inhibition of effector T cell activation and proliferation, suppression of cytokine production and induction of regulatory T cells (114, 117, 118).

Dendritic cells activate and polarize lymphocytes via antigen presentation, a mechanism essential to the clearance of certain infections and prevention of re-infection. Cystatins are a family of relatively non-selective protease inhibitors which may inhibit cathepsins and thereby regulate antigen presentation and T cell activation (119). Tick cystatins alleviate the inflammatory response in the human skin in various forms: *I. scapularis*-derived sialostatin L prevents dendritic cell maturation and subsequent CD4+ T cell proliferation as well as memory formation by inhibition of cathepsin S (120). Additionally, OVA-specific CD4+ T cells showed reduced proliferation upon preincubation of splenocytes with Iristatin and subsequent OVA peptide stimulation.

*I. scapularis* sphingomyelinase-like (IsSMase) protein was shown to increase proliferation of host helper T cells and the expression of the major Th2-cytokine IL-4 in an *in vivo* TCR transgenic adoptive transfer assay (121). *I. ricinus* serine protease inhibitor (IrSPI) was characterized as a Kunitz elastase inhibitor that is overexpressed in tick salivary glands during blood feeding (122). IrSPI inhibited the proliferation of mouse CD4+ T cells *in vitro* and, therefore, suppressed adequate effector T cell response (123). Further, Palenikova et al. (124) could demonstrate that tick saliva serpin IRS-2 inhibits Th17 polarization via the impairment of the IL-6/STAT-3 signaling pathway, and additional mechanism subverting the host immune response to avoid tick rejection (124). Another tick saliva-secreted serpin heavily involved in T cell modulation is Iripin-3. Iripin-3 was shown to decrease CD4+ T cell survival and proliferation and to specifically suppress Th1 immune responses and regulatory T cell (Treg) induction (83). Similarly, the serpins RmS-3 and RmS-17 from *Rhipicephalus microplus* affected metabolic activity and reduced proliferation and IFN-γ production in murine T lymphocytes (125). Additionally, PGE2 in the saliva of these ticks induced PD-1 expression on T cells and PD-L1 expression on CD14+ and CD11c+ cells in cattle and thereby blocked Th1 polarization and cytokine production (126).

Salp15 is one of the most studied tick saliva proteins and has diverse consequences on T cell biology including T cell priming and activation, proliferation and cytokine production (127). It was originally identified as a 15 kDa cysteine-rich glycosylation protein in *I. scapularis* saliva and shown to inhibit CD4+ T cell activation by repressing calcium fluxes triggered by TCR engagement and subsequent DNA binding activity to the transcription factors nuclear factor of activated T cells (NF-AT), and nuclear factor κ B (NF-κB) (117). This resulted in decreased CD25 (IL-2R) expression and IL-2 production by T cells, which play pivotal roles in the adaptive immune response including the regulation of self-tolerance by Tregs and polyclonal T cell activation and proliferation (117, 128, 129). A reduction in the expression of these molecules might facilitate future tick infestations due to a low memory T cell generation, as well as transmission and spreading of tick vectors into the host as a result of impaired pathogen clearance (117, 129, 130). In the meantime, several studies have investigated the various roles of Salp15 in host immune modulation and could show that it binds directly to CD4 on T cells and exerts a long-lasting effect several days after its removal from cultures (131, 132). Thus, Salp15 interferes with T cell receptor (TCR) signaling (133, 134), pro-inflammatory cytokine production, T cell activation by dendritic cells (135), and Th2 effector cell polarization (136). Furthermore, Salp15 induces CD73 expression and adenosine production of Tregs, which consequently repress effector T cell functions (131). Lastly, Salp15 does not only directly interfere with T cell differentiation but also indirectly via antigen-presenting cells. It was shown to bind to the pattern recognition receptor DC-SIGN on dendritic cells and thereby inhibit cytokine production essential for T cell effector functions (135).

In addition to the effects of tick saliva compounds at the cellular level, immunoglobulin-binding proteins (IGBP) lead to a suppression of the host’s humoral immune response by binding immunoglobulins, in particular immunoglobulin G. IGBP have been detected in salivary glands and hemolymph of *R. haemaphysaloides* and salivary gland extract of *R. appendiculatus*, *Amblyomma variegatum* and *I. hexagonus* (137, 138). By binding to immunoglobulins, IGBP enable ticks to evade not only the cellular but also the humoral immune response of their host and thus promote tick feeding. Their presence therefore makes them potential targets for the development of anti-tick vaccines (139).

#### 2.2.1 Influence of tick feeding on B lymphocytes

B cells, which have traditionally been neglected in skin immunity, are now considered to be crucial for the maintenance of skin homeostasis and regulation of immune responses. Although found only in low numbers in healthy human skin, B cells increase in skin adjacent to the bite site upon tick feeding. Upon tick-borne *B. burgdorferi* infection, they display an IgM memory phenotype in erythema migrans skin lesions (73, 140). However, mounting evidence points towards functional impairment of B cells in response to tick saliva.

Salivary gland extract (SGE) from ixodid ticks was shown to decrease CD69 expression on B cells and inhibit their IL-10 production and proliferation in response to LPS stimulation (141). Furthermore, B cell-inhibitor factor derived from *I. ricinus* was demonstrated to inhibit LPS and *B. burgdorferi* lipoprotein-induced B cell proliferation (142). Lastly, the above-described protein Iripin-3, which inhibits T cells, also had a negative effect on B cell viability. Salp15, which inhibits CD4+ T cell activation as mentioned above, also has an indirect effect on antibody production by B cells (117). Mice immunized with Salp15 fused to thioredoxin (TR) showed diminished TR-specific IgG response compared to TR-only immunized animals, which might be explained by the dependency on T cells for Ig class switching in B cells (117).

### 2.3 Consequences of tick saliva-driven cutaneous immune suppression on pathogen transmission

Tick saliva contains many highly specific immunomodulatory compounds that interfere with the function of the innate and adaptive immune response based on the mechanisms mentioned above. The resulting environment not only allows the tick to survive and feed but also promotes the transmission of pathogens. Ticks serve as vectors for a wide variety of pathogens, including viruses, bacteria, and protozoa, facilitating their transmission between hosts (148). Bioactive molecules of tick saliva do not only facilitate immune evasion but also have beneficial effects for tick-borne pathogen replication and survival in vertebrate hosts (149). This phenomenon is called saliva-activated transmission (SAT) and originally defined the promotive effect of SGE of *Rhipicephalus appendiculatus* on Thogoto virus transmission (150). However, in recent years the concept of SAT has been expanded to various tick-borne pathogens and their mechanisms to modify tick saliva composition for their benefit.

#### 2.3.1 Bacterial tick-borne infections

As previously mentioned, Lyme borreliosis is a commonly occurring disease in North America and Europe (151). *B. burgdorferi* s.l. spirochetes have evolved mechanisms to overcome tick barriers, evade tick immune defenses, avoid endocytic digestion, and enhance transmission rate to new hosts (152). *Ixodes* complex saliva protein concentration is increased upon infection with *B. burgdorferi* (153). Hoxmeier et al. (145) identified 114 differentially regulated metabolites between *B. burgdorferi*-infected and uninfected *I. scapularis* nymphs. These metabolites involved in purine, amino acid, carbohydrate and fatty acid metabolism are necessary for bacterial survival and to continue the transmission cycle. *Borrelia* spirochetes lack the capacity for *de novo* synthesis of many of these compounds and sequester them from the blood of vertebrate hosts during blood feeding, contributing to a change in saliva composition of infected ticks (154). Furthermore, *B. burgdorferi* and *Anaplasma phagocytophilum* were shown to induce glycolysis in tick cells (155). β-aminoisobutyric acid was identified to positively contribute to tick fitness and bacterial infection of the tick. The inhibition resulted in diminished survival and reduced bacterial load post-haematophagy. This suggests that a precise β-aminoisobutyric regulation is essential for the initial stages of infection (155).

In addition, changes in the tick metabolism and saliva composition might also have strong impacts on infection susceptibility in their vertebrate hosts. In a murine study, Tang et al. (147) investigated the role of a tick salivary protein, designated *I. scapularis* complement C1q-like protein 3 (IsC1ql3), in tick-transmitted infection. They identified that the gC1q domain of IsC1q13 can directly interact with a *B. burgdorferi* protein ligand, resulting in reduced *B. burgdorferi*-induced IFN-γ production via the interaction with the C1q receptor on CD4+ and CD8+ T cells (156). Tick salivary lectin pathway inhibitor (TSLPI, previously called P8), is a protein that blocks complement-mediated killing of *Borrelia* by interfering with the human lectin complement cascade. TSLPI inhibits complement-mediated recruitment of human neutrophils and phagocytosis of spirochetes by these cells. Further, it was shown that TSLPI is essential for *Borrelia* survival within ticks and that TSLPI-immunized mice had a significantly lower spirochete burden (64). Lymphotoxin beta receptor (LTBR) knockout mice were found to be more susceptible to borrelial spirochetes, and the *I. persulcatus* salivary protein (IpSAP) was identified to bind to LTBR, blocking the downstream signaling cascade, resulting in decreased immune cell infiltration and general immunosuppression. Immunization of mice with IpSAP reduced *I. persulcatus*-mediated *B. garinii* transmission, as well as infection with other *Borrelia* spp. from different ixodid ticks (157). *I. scapularis*-derived Salp15 and its homologues from *I. ricinus* (Iric1, Iric2, Iric3), known for their immunomodulatory capacity, were shown to bind *B. burgdorferi* and *B. garinii* Outer surface protein C (OspC). This direct interaction permits spirochetes to evade antibody-mediated killing by the host immune system and enhances transmission rates (158). *Borrelia burgdorferi*-infected nymphs were identified to secret high concentrations of *I. scapularis* saliva serpin (IxsS41) (159), a serpin highly conserved across *Ixodes* spp (160). Recently, IxsS41 was demonstrated to inhibit pro-inflammatory proteases like chymase, cathepsin G as well as mast cell degranulation compound 48/80, released by activated neutrophils and mast cells. This sufficiently protects *B. burgdorferi* spirochetes from complement-mediated killing by reducing membrane attack complex deposition (160). IxsS17, another highly conserved serpin, inhibits inflammatory effector and regulatory proteases, blood clotting (factor X, plasmin, kallikrein, antithrombin III, heparin cofactor II), but also complement activation (C1s, C2, factor I, plasma protease C1 inhibitor), which results in enhanced *B. burgdorferi* survival, host colonization and transmission (161).

Another compound previously identified in tick saliva are extracellular vesicles (EVs). EVs are nanovesicles that mediate interspecies communication and were recently shown to have immunomodulatory functions during vector transmission (162). *Ixodes scapularis*-derived EVs have the potential to enhance infection with *A. phagocytophilum* during tick feeding on mice, through the SNARE proteins Vamp33 and Synaptobrevin 2 and dendritic epidermal γδ T cells (163). The EVs directly interact with macrophages via integrins and redirect skin immunity by reducing the number of skin resident dendritic epidermal γδ T cells, thereby promoting bacterial infection. Interestingly, when mice were infected with *Francisella tularensis* and EVs of *Dermacentor andersoni*, the opposite effect was observed, resulting in a reduced bacterial burden and higher resistance to infection due to the mitigation of microbial spreading (163). These findings highlight that tick EVs play a significant role in successful bacterial infection.

Overall, due to modulation of T-cell responses by tick saliva proteins, accompanied by a reduction in the CD4+/CD8 T-cell ratio, a shift towards a Th2 response, and impaired T-cell proliferation and cytokine secretion (73, 113, 124, 125) the host may be more susceptible to extracellular pathogens and irritants. Th1 and Th17 cells are essential in the adaptive immune response, exhibiting protective properties against fungi and extracellular bacteria including *B. burgdorferi* spirochetes. Impaired Th1/17 cell function reduces production of associated cytokines, including IFN-γ and may render the host’s skin more susceptible to these pathogens (164–167). In addition, a shift towards Th2 response, which provides defense against helminths and promotes chronic inflammatory conditions, such as allergies (168), may favor the transmission of tick-borne bacterial pathogens including *Borrelia, Anaplasma*, and *Rickettsia* (158, 159).

#### 2.3.2 Viral tick-borne infections

Ticks transmit a great variety of viruses, causing tick-borne encephalitis, Crimean Congo hemorrhagic fever, and Severe Fever with Thrombocytopenia Syndrome (SFTS). Tick saliva has been suggested to facilitate the transmission of these viruses (169).

Thangamani et al. (170) performed RNA sequencing to characterize the early cutaneous immune network in response to *I. ricinus*-transmitted TBEV in BALB/c mice and captured changes in gene expression during the early host immune response (1-3 hours post tick bite). Overall, the genes upregulated 1 hour after tick feeding associated with neutrophil activation and mobilization were mostly downregulated 2 hours later. Together with decreased anti-viral T cell responses, this rapid suppression of the inflammatory cutaneous environment at the tick bite site might enhance viral transmission (170). As more specific mechanism, Tick-borne encephalitis virus (TBEV) was shown to directly interfere with sphingomyelinase D in *I. scapularis* ticks, which allows accumulation of sphingomyelin lipids, beneficial for membrane-associated viral replication and exosome biogenesis. Sphingomyelinase D inhibitor treatment reversed this effect and inhibited viral replication (171). Furthermore, virus propagation in the host might be enhanced by anti-apoptotic effects on TBEV-infected dendritic cells: incubation with *I. ricinus* saliva led to upregulation of PI3K/Akt signaling pathway in infected murine bone marrow-derived dendritic cells (172).

In Crimean Congo hemorrhagic fever virus infection, *Hyalomma marginatum* SGE inhibited the migratory potential of dendritic cells and Langerhans cells, potentially suppressing lymphatic emigration and subsequent adaptive immune responses (173). Wang et al. (168) examined the relationship between the *H. longicornis* saliva peptide HIDfsin2 and the SFTS virus. HIDfsin2 was identified to be beneficial for SFTSV replication in cancer cell lines by MAPK activation in the viral post-entry stage. This effect was specific for SFTSV and could be abrogated by pharmacological blockade of the p38 MAPK cascade, suggesting a potential new therapeutic target (174). On the contrary, in mouse macrophages HIDfsin2 enhanced innate immunity (IL-1β, TNF-α, IL-6, type I IFN pathway) and inhibited SFTSV replication via toll-like receptor (TLR) 4 signaling pathway activation (175). These results highlight the different biological effects of the same peptide in immune and non-immune cells and the need for further investigations of tick saliva proteins in the context of viral transmission and potential therapeutic application.

MicroRNAs (miRNAs) are a non-protein constituent of tick saliva known to counteract host responses. During Powassan virus infection, a severe neuroinvasive disease in humans, 52 miRNAs secreted with the saliva of *I. scapularis* were identified to be differentially regulated during feeding on naïve mice. By transfection of some of these miRNAs into a mammalian cell line, they were characterized as potential regulatory elements for Powassan virus infection and dissemination (176). However, further studies are necessary to identify the exact regulatory mechanisms of miRNAs implicated in viral replication. As miRNAs were demonstrated to be involved in the manipulation and redirection of the host immune response to promote pathogen transmission in other vectors such as mosquitos, in-depth research on these compounds in tick saliva might be promising (177, 178).

#### 2.3.3 Protozoan tick-borne infections

In addition to emerging viral and bacterial diseases, ticks are also vectors of protozoan pathogens, including *Babesia* species. Antunes et al. (169) performed sialotranscriptomic characterization of the salivary glands of *Rhipicephalus bursa* and identified 36 genes differentially regulated in *B. ovis*-infected feeding ticks compared to non-infected feeding ticks. Knock-down experiments using RNA interference of three selected upregulated genes (including *vitellogenin-3*, a secreted cement protein, and *lachesin*) resulted in increased tick mortality, failure of tick attachment and decreased *B. ovis* levels in tick salivary glands (179). While these results highlight the potential modulation of *Babesia* colonization via tick saliva, overall limited literature is available on the influence of tick saliva compounds on protozoan parasite transmission, highlighting the need for research in this area.

## 3 Modulation of the skin tissue microenvironment promoting tick feeding efficiency

### 3.1 Tick saliva suppresses nociception

**Figure 2:**
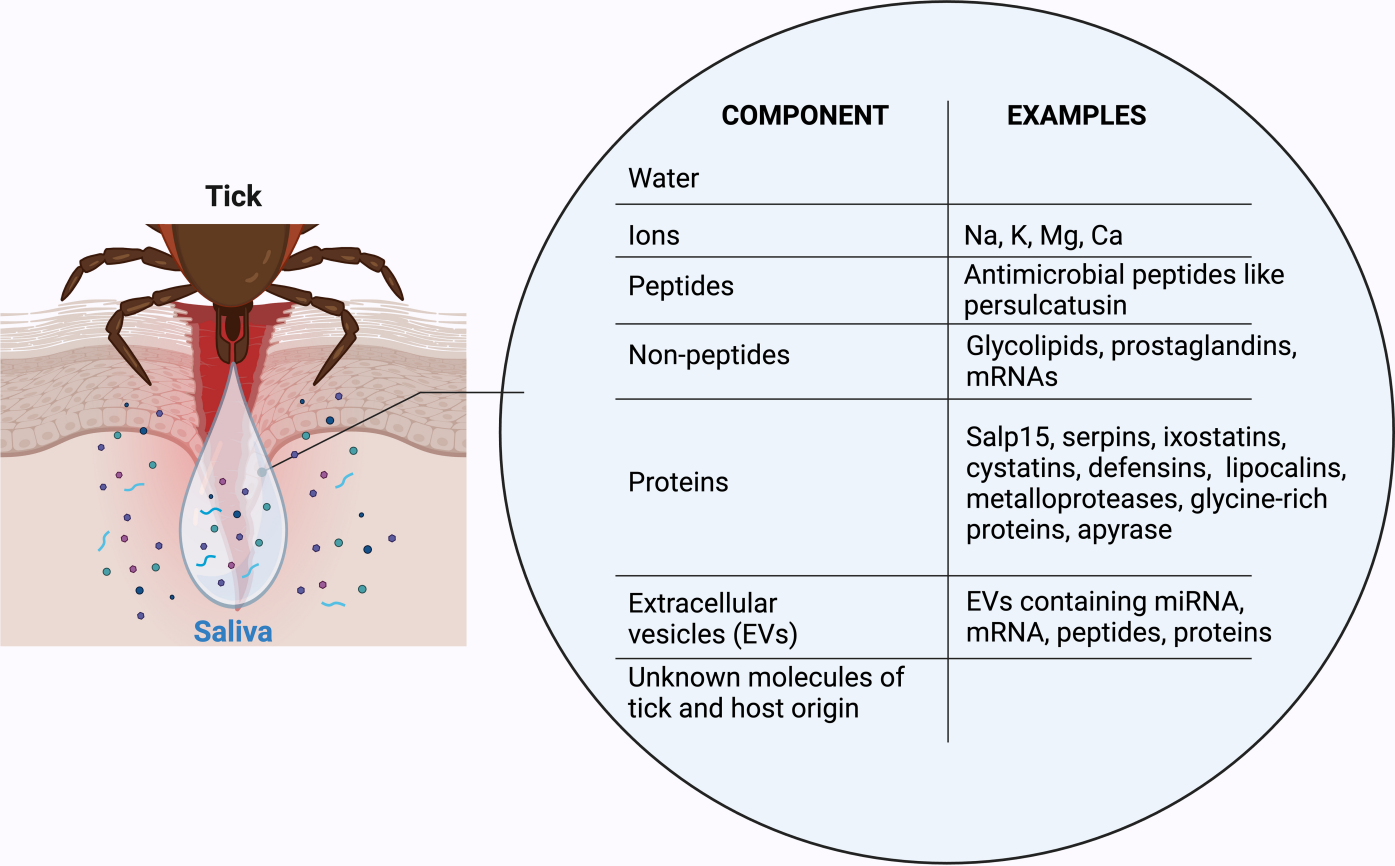
Tick saliva components influence cutaneous immunity, wound healing, vasculature and nerve fibers. Tick saliva inhibits innate and adaptive immunological functions by reduction of neutrophil and mast cell function, impairment of immunogenic antigen presentation and increasing the proportion of Th2 cells in human skin. Tick saliva molecules mediate endothelial damage and increased permeability, and inhibit angiogenesis, blood clot formation and disruption of fibrinolysis. Additionally, tick saliva components have inhibitory effects on ECM remodeling and degradation and keratinocyte (KC) re-epithelialization in wound beds.

The sensations of itch and pain are protective mechanisms that alert the individual to potential danger or environmental irritants, leading to a behavioral response, including scratching (180, 181). Once the tick’s mouthparts penetrate the skin, several mediators are released that are involved in the activation of sensory nerve fibers leading to nociception. However, tick saliva components have been found to suppress cutaneous nociception (**Table 3**; **Figure 2**) (68).

**Table 3:** Putative effects of tick saliva components on blood vessels, nerve fibers and extracellular matrix (ECM) in the skin.

Blood vessels	Nerve fibers	ECM
Inhibition of angiogenesis	Suppression of nociception	Inhibition of wound healing
Damage of endothelial integrity	Inhibition of neurotransmitters	Altered degradation of extracellular matrix proteins
Inhibition of thrombin-regulated vessel modulations	Degradation of pain mediators	Dysregulated extracellular matrix remodeling
Increased vascular permeability	Counteraction of endocannabinoid-induced immunosuppression	Impaired keratinocyte migration

The release of neurotransmitters, including histamine and serotonin, by mast cells and basophils is associated with the development of both pain and itch. These mediators are counteracted by tick saliva components, such as tick histamine binding proteins (tHBP) from *I. scapularis* (182), serotonin-histamine binding protein (SHBP) in *Dermacentor reticulatus* ticks (67, 183), *R. appendiculatus*-histamine binding proteins (Ra-HBPs) (67, 144, 184), and HA24 from *Hyalomma asiaticum* ticks (67, 185).

Another cutaneous pain meditator, bradykinin, is a kinin peptide released in injured skin that acts on receptors located on terminal sensory nerve fibers (186–191). Metalloproteases present in *I. scapularis* saliva degrade bradykinin and counteract this nociceptive pathway (192, 193).

In addition, tick saliva and salivary glands contain high concentrations of arachidonic acid, which is a precursor to a range of signaling molecules involved in nociception. During the blood meal, modifications in the arachidonic acid metabolism occur (32, 194). The arachidonic acid derivates endocannabinoids are lipid neuropeptides with a regulatory effect on pain, nociception and inflammation. They act on cannabinoid receptors 1 and 2 expressed on nerves and immune cells, including T/B lymphocytes and monocytes (195–197). These leukocytes were described to form clusters in proximity to sensory nerves and communicate via soluble factors with nerve fibers, regulating inflammatory and sensory pathways (198, 199). Fezza et al. (32) investigated the role of endocannabinoids during tick feeding and suggested that these lipids are produced in tick salivary glands mainly in response to an external stimulus with arachidonic acid contributed by the host. Thus far, these experiments have only been conducted on saliva derived from the lone star tick *Amblyomma americanum.* Further investigations are necessary in order to assess the presence, abundance and function of endocannabinoids in the *Ixodes* genus. Additionally, the impact of these compounds on nociception in the host should be considered. This is particularly important given that the interaction of the nervous system with the immune system impacts homeostasis and host responses to external stimuli in the skin.

### 3.2 Tick saliva counteracts hemostasis

Hemostasis is a physiological cascade responsible for the stasis of bleeding in response to injury. It involves primary hemostasis with vasoconstriction and platelet aggregation and secondary hemostasis, resulting in fibrin formation creating the final platelet plug (200). To ensure blood flow during feeding, tick saliva provides mechanisms to inhibit coagulation (**Figure 2**). Primary hemostasis is initiated by platelets binding to endothelial cells, followed by platelet activation with adenosine diphosphate as a crucial activator (201). Multiple components in tick saliva have been found to target different steps of platelet activation (30). The enzymatic component Apyrase inhibits platelet activation by hydrolyzing adenosine triphosphate, adenosine diphosphate to adenosine monophosphate, and inorganic phosphate incapable of platelet activation (201). Other components, including serpins and antithrombins, target thrombin and cathepsin G, which both lead to platelet aggregation (202–204). Secondary hemostasis by intrinsic and extrinsic coagulation systems results in the activation of factor X, which converts prothrombin into thrombin, leading to fibrin formation (200). Many factor X inhibitors have been detected in *Ixodes* tick saliva, including tick inhibitor of factor Xa toward factor V (TIX 5) (205), Salp14 (35), *I. ricinus* immunosuppressor (Iris) (206), Ixolaris (207–209), *I. ricinus* contact phase inhibitor (Ir-CPI) (210), *I. ricinus* serpin-2 (IRS-2) (211) and *I. scapularis* tick saliva serine protease inhibitor (IxscS-1E1) (210). Many of the listed effectors additionally target other steps of the coagulation cascade as well as different stages of wound healing. For example, Iris is not only a specific elastase inhibitor that controls fibrinolysis but also has anti-inflammatory and immune-modulating effects (206, 212, 213). Although numerous proteins and their role in hemostasis have been identified in the past few decades, a considerable number of proteins with essential roles in vector-host interactions remain to be discovered.

In addition to its effects on coagulation, tick saliva directly affects the vasculature at the skin interface by modulation of endothelial cell proliferation, vessel permeability and angiogenesis. (Neo-)angiogenesis is the development of new blood vessels from pre-existing ones and occurs during homeostasis, inflammation and in late phases of wound healing (214, 215). Francischetti et al. (216) demonstrated that the saliva of *I. scapularis* has inhibitory effects on endothelial cell proliferation and angiogenesis. They identified that it inhibits the proliferation of microvascular endothelial cells *in vitro* and suggested metalloproteases as responsible enzymes. Additionally, they found tick saliva to cause cell shrinkage, endothelial integrin degradation and apoptosis (**Figure 2**) (216).

In a recently published paper, Berger et al. (217) characterized persulcatin present within tick saliva of *I. persulcatus* and highlight the importance of persulcatin in a variety of early cutaneous responses following tick bites. Persulactin is an inhibitor of the enzymes plasmin, involved in hemostasis and wound healing, and thrombin, responsible for blood clot formation. It influences tight junctions of endothelial cells via inhibition of thrombin-regulated vessel modulations and thus decreases vascular permeability, which would normally promote leukocyte migration and inflammation (217).

Our review is primarily focused on the impact of tick saliva on immunomodulation, for a detailed discussion of the effects on hemostasis we recommend the following reviews by Chmelar et al. (218), Francischetti et al. (219) and Jmel et al. (220).

### 3.3 Implications of tick saliva for wound healing

The process of wound healing is a multiphase phenomenon that encompasses the regulation of hemostasis, inflammation, cell proliferation and migration, and tissue remodeling (221, 222). These phases collectively present a challenge for ticks to stay attached to their hosts and achieve a successful blood meal (217). Nevertheless, ticks benefit from the effects of certain salivary compounds, which modulate wound healing processes in the host skin (**Figure 2**) (223). Zhou et al. (224) isolated small extracellular vesicles from ixodid saliva and salivary glands and demonstrated an inhibitory effect on wound healing: When treating keratinocytes with these exosomal vesicles, an increase in interleukin (IL)-8 expression and, simultaneous decrease in CXCL-12 was observed, resulting in impaired cell migration and consequently impaired tissue repair (224). Tick salivary metalloproteases are believed to promote ECM degradation (225). However, the Kunitz-type protease inhibitor persulactin – mentioned above in the context of coagulation - has also been shown to impede keratinocyte migration, and the degradation of extracellular matrix proteins by inhibiting matrix metalloproteinase 2 and 9 expression by keratinocytes (217). While keratinocytes play a pivotal role in wound healing, to date their involvement in the context of tick bites has received little attention and further research is required to elucidate the major effects of tick saliva compounds on keratinocyte migration and function.

### 3.4 Tick saliva compounds alter the skin microbiome

The function of tick intestinal microbiota in disease transmission and pathogenesis has been the subject of several studies. However, the role of the host skin microbiota in this context is not yet well understood (26). The human skin microbiome, comprising, among others, viruses, fungi and bacteria, plays an essential role in maintaining skin homeostasis and is part of the first line of defense against invading pathogens (226–228). The composition of bacteria colonizing the skin is influenced by the anatomical location and varies across different human body sites. The most prevalent species on the cutaneous surface include *Staphylococcus, Corynebacterium*, and *Cutibacterium* (229, 230). During inflammatory processes, the composition of the cutaneous microbiota can be altered. However, the specific effects of pathogens and vectors remain to be identified (231, 232). The microbiome exhibits intimate contact with keratinocytes, given that these cells constitute the outermost skin layer. Baquer et al. (233) used an *in vitro* co-incubation model to demonstrate the antimicrobial effect of keratinocyte-microbiome interaction in promoting an early immune response to *B. burgdorferi* infection using secretomes of *Staphylococcus epidermidis, Corynebacterium striatum*, and *Cutibacterium acnes*. The authors further suggest a pro-inflammatory reaction of the skin microbiome towards tick bites. However, bacterial clearance in this regard could not be determined in this model (233). Interestingly, tick bites, especially of those in the nymph stage, significantly alter the murine skin microbiome, including replacement of commensals by tick-borne bacteria such as *Borrelia* spp (234). The impact of tick feeding on the human cutaneous microbiome and consequences of microbiome alterations on host-pathogen interactions have not been investigated. On the other hand, it is plausible that unaltered commensal microbiota may contribute to tick-directed immune responses in the skin and thus influence the pathophysiology of tick-borne diseases. Furthermore, the development of tick-borne vaccines may benefit from a more comprehensive understanding of the complex interactions between the host, microbiome, vector, and pathogen (231).

## 4 Tick saliva proteins as potential tick vaccine immunogens

Given the fact that tick territories are expanding worldwide, the incidence of tick-borne diseases is increasing, and the numbers of newly identified tick-borne pathogens are rising, there is an urgent need for a broad anti-tick vaccine (235, 236). Successful vaccines exist for few tick-borne diseases like TBE, but for others, such as Lyme borreliosis, they do not. Therefore, current research focuses on tick saliva compounds as potential vaccine candidates to reduce the overall tick burden and transmission of tick-borne illnesses, as outlined by Boulanger & Wikel 2023 and Johnson et al., 2024 (237, 238).

To identify novel proteins serving as potential vaccine antigens, liquid chromatography can generate tick saliva fractions to screen for their tick immunity induction ability. Using mass spectrometry, Černy et al. (226) identified proteins associated with tick resistance, which included serpins, *I. scapularis*-derived antimicrobial proteins (microplusin, ricinusin, hebraein), and leucine-rich repeat proteins (239).

To create a global anti-tick vaccine, Sajid et al. (226) immunized guinea pigs with lipid nanoparticle–containing nucleoside-modified mRNAs encoding 19 *I. scapularis* salivary proteins (19ISP). When animals were subsequently challenged with *I. scapularis*, they rapidly developed visible skin inflammation, suggesting immune cell infiltration, and attached ticks fed poorly. The antibody response was boosted by 19ISP immunization and *B. burgdorferi* transmission was impeded, suggesting potential application as a novel vaccine candidate (240). In a different study, immunization of Kunming mice with three generated recombinant serpins conserved in *Rhipicephalus* spp. resulted in a drastic reduction of successful tick engorgement after challenge with *R. sanguineus*, indicating a potential use in the development of a universal anti-tick vaccine (241). Costa et al. (242) investigated three *Amblyomma sculptum* proteins: the Kunitz bovine pancreatic trypsin inhibitor domain (AsKunitz) protein, the 8.9 kDa protein and basic tail families of tick salivary proteins (As8.9kDa, AsBasicTail), all of which are present in increased levels in feeding ticks. They inhibit the enzymatic activity of factor Xa and thrombin, as well as hemolysis of the classical and alternative complement system. Immunization of mice with these recombinant proteins led to an increase in specific IgG levels, and mortality and egg hatching of engorged ticks were reduced (242). Kotsyfakis et al. (243) pre-sensitized guinea pigs to Sialostatin L2 using high amounts of this protein in a vaccine, resulting in compromised blood feeding by *I. scapularis*. Thus, they showed that although sialostatin L2 typically evades immune detection, it can be targeted for immune intervention when presented in sufficient quantities (243).

In a recent study, cellular and humoral immune responses against *Hyalomma anatolicum* were induced using two multi-epitopic peptides. Immunization efficacy in rabbits was > 90% in larvae and >85% in *H. anatolicum* adult ticks after vaccination with these peptides. Both peptides were sufficient to induce a significant increase in IgG levels and a decrease in the secretion of the anti-inflammatory cytokine IL-4. Immunization with one of the peptides increased the production of pro-inflammatory IL-2 (244). While these findings highlight the immense potential of tick-derived saliva molecules to be used as global anti-tick vaccines, the design of human tick-vaccination studies is challenging, and several potential risks and side effects will have to be excluded before broad applicability.

## 5 Conclusion and future perspectives: research on tick salivary proteins and their use as novel therapeutics

Tick saliva research is a rapidly evolving field with potential for numerous future developments. Promising areas for novel discoveries include the deep characterization of salivary compounds using advancements in proteomic and transcriptomic profiling. These steadily evolving techniques may help identify novel proteins, peptides, microRNAs and other molecules in tick saliva with unknown functions. In addition, advancements in structural biology can be used to determine the three-dimensional structures of key salivary proteins to elucidate binding potential and hint towards their mechanisms of action. Synergistically, one may utilize machine learning, to predict the roles of specific salivary molecules in immune evasion and pathogen transmission. These predictions will need to be confirmed by functional studies, for example, by using organoid systems to reduce the use of animal models.

*Ex vivo* and *in vitro* systems may also help to elucidate mechanisms of cross talk to understand how tick saliva modulates the host’s microbiome and skin immune response to create a favorable environment for pathogen transmission. Many of these mechanisms are likely pathogen-specific and will thus need to be investigated in the context of different bacterial and viral species. However, knowledge of interactions at the tick-pathogen-host interface is pivotal for the development of tick and pathogen-directed vaccines.

Another promising field of research is the repurposing of tick salivary proteins as templates for novel drugs. Several bioactive compounds have been isolated from tick saliva, which may be useful for the development of novel therapeutics for a broad range of diseases. Protease inhibitors, including Kunitz-type inhibitors (208, 209, 245), cystatins (143, 246), and serpins (212) have constituted a focus of considerable interest in recent years, with numerous tick saliva research projects devoted to them (247). Wu et al. (248) recently demonstrated the therapeutic potential of tick protease inhibitors, including Iristatin and Sialostatin L/ L2 in psoriasis, a prevalent chronic inflammatory skin disease. The authors propose cutaneous application of these inhibitory molecules as an alternative approach to systemic administration, suggesting neutralizing effects against key cytokines of psoriatic inflammation and alleviation of symptoms with few side effects (248).

Another promising candidate for therapeutic repurposing is *Ornithodoros moubata* Complement Inhibitor (OmCI), which inhibits the complement cascade by interaction with protein C5, and was shown successful in treating sepsis in animal models (249–251).

Furthermore, Salp15 is frequently described as a candidate for treating autoimmune diseases, such as systemic lupus erythematosus (127, 252). Salp15 was shown to be effective in the treatment of murine graft-versus-host disease, a severe immunological complication after allogeneic hematopoietic stem cell transplantation driven by donor CD4^+^ T cells (118, 127, 253, 254). In mouse models, Salp15 exhibited protective effects by promoting CD4^+^ suppression and prevention of immune-complex damage to the kidneys (118, 127). Additionally, Salp15 has been shown to improve symptoms of allergic asthma in mice by inhibiting Th2, inflammatory cytokines and airway hyper-responsiveness (127, 255, 256). Although many beneficial effects of Salp15 have been discovered in the treatment of several experimental diseases, pre-clinical studies are required to determine potential adverse events and the efficacy of these drug candidates in humans (127).

In the last few years, non-coding RNAs, including miRNAs, in tick saliva have emerged as a significant area of investigation as they are taken up by host cells, where they regulate gene expression and influence the interaction between hosts, pathogens and vectors (257, 258). The ability of miRNAs to modulate host gene expression to mediate transcriptional control of homeostatic processes within the host while providing low immunogenicity illustrates their potential for therapeutic application and is an active area of research (259).

Overall, tick species can mediate an astonishing variety of “tricks” to promote their blood meal at the cutaneous interface of their respective host. While these fascinating mechanisms are certainly evolutionary advantageous for ticks, they lead to increased risk for tick-borne infections of the host. However, the therapeutic repurposing of ticks’ tricks to combat human disease is a promising, newly expanding field in immunological research. Future directions in tick saliva research are likely to benefit from interdisciplinary approaches, integrating insights from molecular biology, immunology, ecology, and computational biology. Progress in these areas could lead to breakthroughs in combating tick-borne diseases and leveraging tick saliva for medical and scientific advancements.
